# Role of Pancreatic Transcription Factors in Maintenance of Mature β-Cell Function

**DOI:** 10.3390/ijms16036281

**Published:** 2015-03-18

**Authors:** Hideaki Kaneto, Taka-aki Matsuoka

**Affiliations:** 1 Department of Diabetes, Endocrinology and Metabolism, Kawasaki Medical School, 577, Matsushima, Kurashiki 701-0192, Japan; 2 Department of Metabolic Medicine, Osaka University Graduate School of Medicine, Osaka 565-0871, Japan; E-Mail: matsuoka@endmet.med.osaka-u.ac.jp

**Keywords:** pancreatic β-cells, oxidative stress, PDX-1, MafA, GLP-1

## Abstract

A variety of pancreatic transcription factors including PDX-1 and MafA play crucial roles in the pancreas and function for the maintenance of mature β-cell function. However, when β-cells are chronically exposed to hyperglycemia, expression and/or activities of such transcription factors are reduced, which leads to deterioration of β-cell function. These phenomena are well known as β-cell glucose toxicity in practical medicine as well as in the islet biology research area. Here we describe the possible mechanism for β-cell glucose toxicity found in type 2 diabetes. It is likely that reduced expression levels of PDX-1 and MafA lead to suppression of insulin biosynthesis and secretion. In addition, expression levels of incretin receptors (GLP-1 and GIP receptors) in β-cells are decreased, which likely contributes to the impaired incretin effects found in diabetes. Taken together, down-regulation of insulin gene transcription factors and incretin receptors explains, at least in part, the molecular mechanism for β-cell glucose toxicity.

## 1. Role of Pancreatic Transcription Factors in the Pancreas

**Figure 1:**
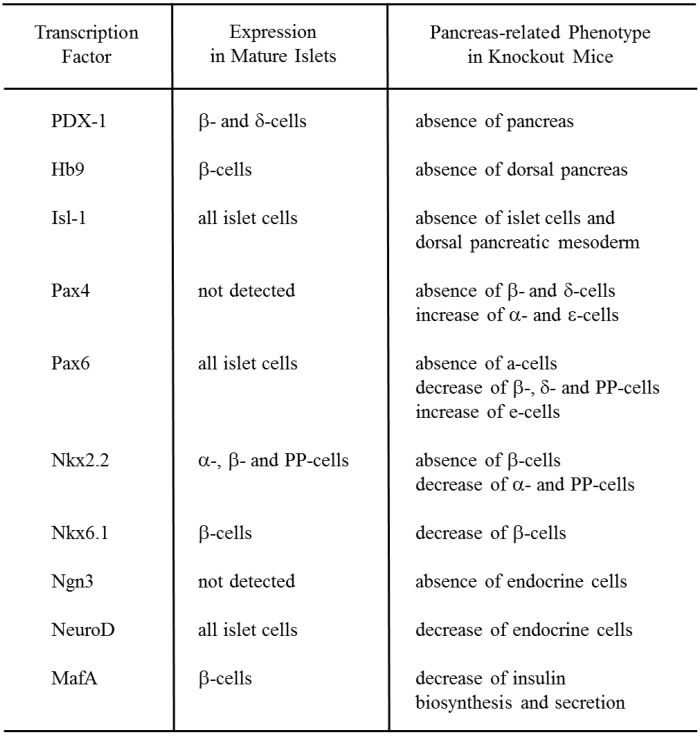
Pancreas-related phenotype in knockout mice of each pancreatic transcription factor.

The adult pancreas is composed of exocrine (acini and ducts) and endocrine compartments (α-, β-, δ-, ε-, and PP-cells). Each of the five endocrine cell types synthesizes and secretes one hormone: glucagon (α-cells), insulin (β-cells), somatostatin (δ-cells), ghrelin (ε-cells), and pancreatic polypeptide (PP-cells). It has been shown that various pancreatic transcription factors are involved in pancreas development and β-cell differentiation. Pancreatic and duodenal homeobox factor-1 (PDX-1) (also known as IDX-1/STF-1/IPF1) [1,2,3] and Hb9, both of which are members of the large family of homeodomain (HD)-containing proteins, play a crucial role in the early stage of pancreas development. While PDX-1 affects the development of the entire pancreas [4,5,6,7,8,9,10,11,12,13], Hb9 plays an important role for the development of the dorsal pancreas [14,15] (Figure 1). Other subclasses of homeodomain (HD) proteins such as Arx, the LIM domain protein Isl-1, the paired domain proteins Pax4 and Pax6, and the Nkx class proteins Nkx6.1 and Nkx2.2 also play an important role in pancreas development [16,17,18,19,20,21,22,23,24,25,26,27]. Pancreas-related phenotype in knockout mice of each homeodomain protein is as follows: Arx (−/−), absence of α-cells and increase of β- and δ-cells [27]; Isl-1 (−/−), absence of islet cells [16]; Pax4 (−/−), absence of β-cells, decrease of δ-cells, and increase of α- and ε-cells [17,24]; Pax6 (−/−), absence of α-cells, decrease of β-, δ- and PP-cells, increase of ε-cells [18,19,25]; Nkx6.1 (−/−), decrease of β-cells; Nkx2.2 (−/−), absence of β-cells, decrease of α- and PP-cells, and increase of ε-cells [20,21,24] (Figure 1). In addition, it is noted that Arx and Pax4 are up-regulated in Pax4 (−/−) and Arx (−/−) mice, respectively, in endocrine precursor cells, and thereby these two transcription factors are likely to play opposite roles for proper endocrine specification [27].

**Figure 2:**
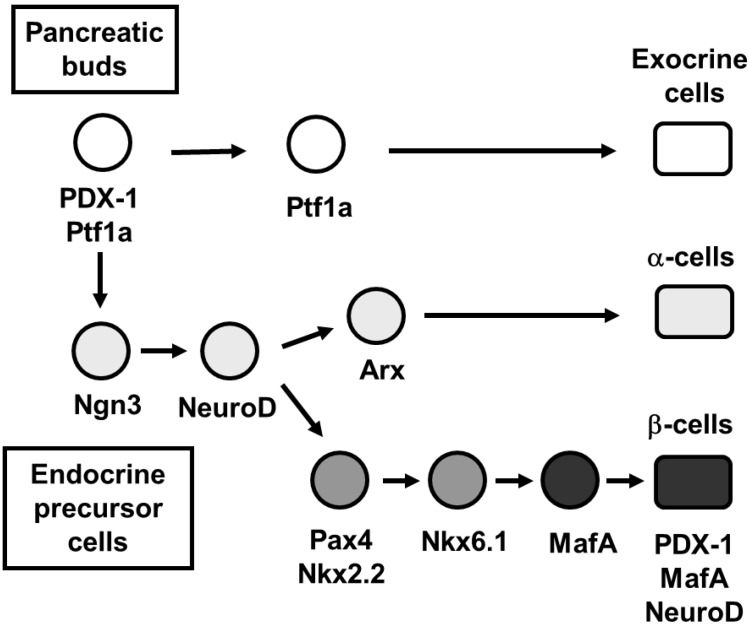
Pancreatic transcription factor hierarchy during pancreas development. It is well known that many transcription factors are involved in pancreas formation and β-cell differentiation. Among the various transcription factors, PDX-1 plays a crucial role in pancreas formation and β-cell differentiation, and maintenance of mature β-cell function. Ngn3 and NeuroD are also important transcription factors for pancreatic endocrine cell differentiation. MafA expression is induced at the final stage of β-cell differentiation and functions as a potent activator of insulin gene transcription.

It is well known that PDX-1 plays a crucial role in pancreas development [4,5,6,7,8,9,10,11,12], β-cell differentiation [28,29,30,31,32,33,34,35,36,37,38], and maintenance of mature β-cell function by regulating several β-cell-related genes [39,40,41,42,43,44,45,46,47]. At an early stage of embryonic development, PDX-1 is initially expressed in the gut region when the foregut endoderm becomes committed to common pancreatic precursor cells. PDX-1 expression is maintained in precursor cells during pancreas development but becomes restricted to β-cells in mature pancreas (Figure 2). In mice homozygous for a targeted mutation in PDX-1, pancreas formation is not observed [4], indicating that PDX-1 plays a crucial role for the formation of endocrine and exocrine cells. Loss of PDX-1 function resulted in pancreatic agenesis in humans as well as in mice [9]. Differentiation and maintenance of the β-cell phenotype also requires PDX-1. In mature β-cells, PDX-1 transactivates the insulin gene and other genes involved in glucose sensing and metabolism such as GLUT2 and glucokinase [42,43]. It was also reported that PDX-1 (+/−) mice were glucose intolerant, with increased islet apoptosis, a decreased islet mass, and abnormal islet architecture, indicating that gene dosage for PDX-1 is crucial for normal glucose homeostasis [10,43,45]. These findings are concordant with the report that humans heterozygous for an inactivating mutation of PDX-1 cause maturity-onset diabetes in the young (MODY 4) [48]. It is noted here, however, that dominant monogenic MODY4 mutations in the PDX-1 gene in humans are not necessarily equivalent to PDX-1 heterozygosity in mice or humans, because it has been reported that recessive mutations in the PDX-1 gene also lead to susceptibility to Type 2 diabetes in humans [49,50,51,52]. Furthermore, to explore a role of PDX-1 in the formation and maintenance of the pancreas, genetically engineered mice were developed using the Tet-off system so that the only source of PDX-1 is a transgene that can be controlled by tetracycline or doxycycline [12]. Since in these mice the coding region of the endogenous PDX-1 gene is replaced with that for the tetracycline-regulated transactivator (tTA), in the absence of doxycycline, tTA activates the transcription of a transgene encoding PDX-1. Expression of the transgene-encoded PDX-1 rescued the PDX-1-null phenotype, and doxycycline-mediated repression of the transgenic PDX-1 throughout gestation recapitulated the PDX-1 null phenotype. Doxycycline treatment at mid-pancreogenesis blocked further development [12]. Also, when PDX-1 expression was shut off with doxycycline in adult mice, insulin biosynthesis was decreased and glucose homeostasis was disturbed [12]. These data further strengthen the importance of PDX-1 in pancreas development, β-cell differentiation, and maintenance of mature β-cell function.

The other well-represented class of transcription factors is that of the basic helix loop helix (bHLH) proteins, which include NeuroD and neurogenin3 (Ngn3). NeuroD, a member of the bHLH transcription factor family, also known as BETA2, is expressed in pancreatic and intestinal endocrine cells and neural tissues. NeuroD plays an important role in pancreas development and in regulating insulin gene transcription [53,54,55,56]. Mice homozygous for the null mutation in NeuroD have a striking reduction in the number of β-cells, develop severe diabetes and die perinatally [57] (Figure 1). Furthermore, it has been reported that the insulin enhancer elements, E-box (NeuroD binding site) and A-box (PDX-1 binding site), are very important for insulin gene transcription [57,58]. Neurogenin3 (Ngn3), a member of the basic helix-loop-helix (bHLH) transcription factor family, is involved in endocrine differentiation [59,60,61,62,63,64,65]. After bud formation, Ngn3 is transiently expressed in endocrine precursor cells, and functions as a potential initiator of endocrine differentiation. Transgenic mice overexpressing Ngn3 show a marked increase in endocrine cell formation, indicating that Ngn3 induces islet cell precursors to differentiate [60,61]. In contrast, mice with targeted disruption of Ngn3 have no endocrine cells [62] (Figure 1).

It was known that an unidentified β-cell-specific nuclear factor bound to a conserved *cis*-regulatory element called RIPE3b1 in the insulin gene enhancer region and functioned as an important transactivator for the insulin gene [66,67]. This important transactivator was identified as MafA, a basic-leucine zipper (bLZ) transcription factor [68,69,70]. MafA controls β-cell-specific expression of the insulin gene through a *cis*-regulatory element called RIPE3b1 and functions as a potent transactivator for the insulin gene [68,69,70,71,72,73,74,75]. During pancreas development, MafA expression is first detected at the beginning of the principal phase of insulin-producing cell production while other important transcription factors such as PDX-1 and NeuroD are expressed from the early stage of pancreas development (Figure 2). In addition, while both PDX-1 and NeuroD are expressed in various cell types in islets, MafA is expressed only in β-cells and functions as a potent activator of insulin gene transcription. Thus, the potency of MafA as an insulin gene activator, together with its unique expression in β-cells, raises the likelihood that MafA is the principal factor required for β-cell formation and function. Furthermore, it was reported that MafA knockout mice displayed glucose intolerance and developed diabetes mellitus [72]. In MafA (−/−) mice, expression of insulin 1, insulin 2, PDX-1, NeuroD, and GLUT2 was decreased, and glucose-, arginine-, and KCl-stimulated insulin secretion was severely impaired (Figure 1). The MafA (−/−) mice also displayed age-dependent pancreatic islet abnormalities [72]. These results strengthen the importance of MafA in the maintenance of mature β-cell function.

## 2. Involvement of Pancreatic Transcription Factors in β-Cell Glucose Toxicity

It is well known that pancreatic β-cells secrete insulin when blood glucose levels become high. However, when β-cells are chronically exposed to hyperglycemia, β-cell function gradually deteriorates, which leads to the aggravation of type 2 diabetes. Once hyperglycemia becomes apparent, β-cell function such as insulin biosynthesis and secretion progressively deteriorates. This process is known as β-cell glucose toxicity which is often observed under diabetic conditions. In the diabetic state, hyperglycemia *per se* and subsequent induction of oxidative stress decrease insulin biosynthesis and secretion and finally bring about apoptosis [76,77,78,79,80,81,82,83,84,85,86,87,88,89,90,91,92,93,94,95,96,97].

**Figure 3:**
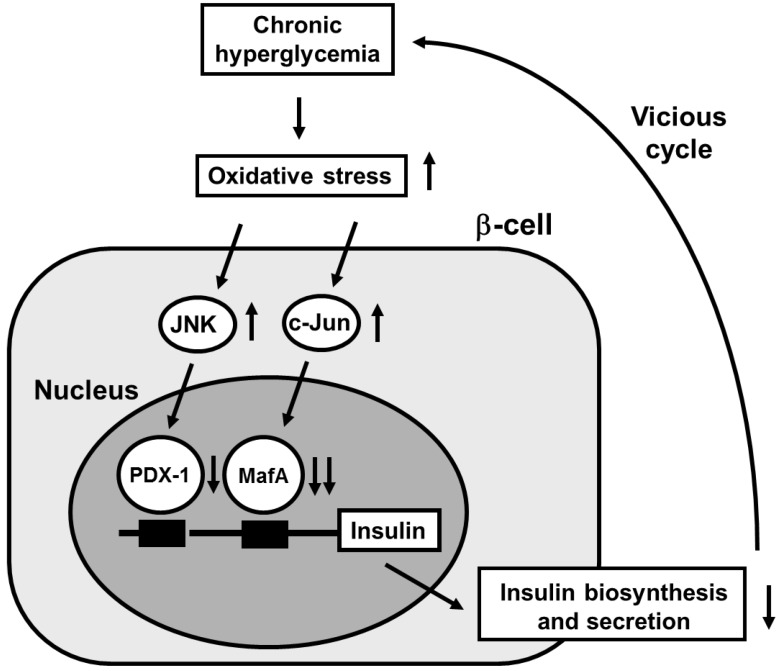
Possible molecular mechanism for suppression of insulin biosynthesis in type 2 diabetes. Under diabetic conditions, hyperglycemia induces oxidative stress and thereby leads to suppression of insulin biosynthesis and secretion, which is accompanied by reduction of nuclear PDX-1 and MafA expression. Therefore, it is likely that induction of oxidative stress and suppression of nuclear PDX-1 and MafA expression are involved in β-cell glucose toxicity found in type 2 diabetes.

Under diabetic conditions, oxidative stress is induced through various pathways and involved in β-cell glucose toxicity. β-Cells express GLUT2, a high-Km glucose transporter, and thereby display highly efficient glucose uptake when exposed to a high glucose concentration. Indeed, it was shown that expression levels of oxidative stress markers such as 8-hydroxy-2'-deoxyguanosine (8-OHdG) and 4-hydroxy-2,3-nonenal (4-HNE) were increased in islets under diabetic conditions [81,83]. In addition, β-cells are rather vulnerable to oxidative stress due to the relatively low expression of antioxidant enzymes such as catalase, and glutathione peroxidase. Therefore, it is likely that oxidative stress is involved in the deterioration of β-cell function found in diabetes. It was shown that when β-cell-derived cell lines or isolated islets were exposed to oxidative stress, insulin gene promoter activity and mRNA expression were suppressed [87,88,89,90,91,92,93,94,95,96]. In addition, when they were exposed to oxidative stress, bindings of pancreatic transcription factors PDX-1 and/or MafA to the insulin gene promoter were reduced. Furthermore, it was shown that the decrease of insulin gene expression after chronic exposure to a high glucose concentration was prevented by treatment with antioxidants [81,86,92,93]. Reduction of expression and/or DNA binding activities of PDX-1 and/or MafA by chronic exposure to high glucose was also prevented by antioxidant treatment. These results suggest that chronic hyperglycemia suppresses insulin biosynthesis and secretion by increasing oxidative stress, accompanied by reduction of expression and/or DNA binding activities of two important pancreatic transcription factors PDX-1 and MafA. Therefore, it is likely that the alteration of such transcription factors explains, at least in part, the suppression of insulin biosynthesis and secretion, and thereby is involved in β-cell glucose toxicity (Figure 3).

It has been suggested that activation of the c-Jun *N*-terminal kinase (JNK) pathway is involved in pancreatic β-cell dysfunction found in type 2 diabetes. It was reported that activation of the JNK pathway is involved in reduction of insulin gene expression by oxidative stress and that suppression of the JNK pathway can protect β-cells from oxidative stress [96]. When isolated islets were exposed to oxidative stress, the JNK pathway was activated, preceding the decrease of insulin gene expression. Adenoviral overexpression of dominant-negative type JNK1 (DN-JNK) protected insulin gene expression and secretion from oxidative stress. These results were correlated with change in the binding of PDX-1 to the insulin gene promoter. Adenoviral overexpression of DN-JNK preserved PDX-1 DNA binding activity in the face of oxidative stress, while wild type JNK overexpression decreased PDX-1 DNA binding activity [96]. Taken together, it is likely that activation of the JNK pathway leads to decreased PDX-1 activity and consequent suppression of insulin gene transcription found in the diabetic state. Also, it was shown that PDX-1 was transported from the nuclei to the cytoplasm in response to oxidative stress [97]. Suppression of the JNK pathway inhibited the oxidative stress-induced PDX-1 translocation, suggesting that activation of the JNK pathway is involved in PDX-1 translocation by oxidative stress. Taken together, it is likely that oxidative stress induces nucleo-cytoplasmic translocation of PDX-1 through activation of the JNK pathway, which leads to reduction of its DNA binding activity and suppression of insulin biosynthesis (Figure 3).

It is known that c-Jun protein level and activity are increased in response to oxidative stress in various cells [98,99]. We recently reported that c-Jun expression was not clearly detected in islets of control *m*/*m* mice, but that the number of c-Jun-positive cells gradually increased with age in the islets of diabetic *db*/*db* mice [100]. This expression pattern of c-Jun paralleled the loss of insulin gene transcription factor MafA expression; while c-Jun mRNA level was significantly increased, both MafA and insulin mRNA levels were markedly decreased with age [100]. These results imply that the increased level of c-Jun caused a decrease in MafA and insulin gene expression in old diabetic mice. Furthermore, in immunostaining, in *db*/*db* mice nuclear MafA expression in pancreatic islets was markedly decreased with age and was not clearly detected in old mice [100]. In *db*/*db* mice insulin expression was also decreased in some cells in which MafA was undetectable or weakly expressed. Furthermore, MafA and insulin expression was suppressed in most c-Jun-positive cells. Similarly, in islets of diabetic KKAy mice, the number of c-Jun-positive cells was increased with marked hyperglycemia, and both MafA and insulin protein levels were decreased in those cells [100]. These findings suggest that c-Jun is involved in the suppression of MafA and insulin expression under diabetic conditions. In addition, c-Jun overexpression markedly decreased insulin promoter activity, which was consistent with previous reports [101,102] (Figure 3).

Although c-Jun protein expression was almost undetectable in MIN6 cells, adenoviral c-Jun overexpression markedly suppressed MafA protein level and its DNA-binding activity in MIN6 cells [100]. Adenoviral overexpression of c-Jun in isolated mouse islets also markedly suppressed MafA mRNA and protein levels. Consistent with these results, insulin mRNA and protein levels were suppressed by c-Jun overexpression in both MIN6 cells and islets [100]. These findings directly demonstrate that c-Jun suppresses the expression of both MafA and insulin. In addition, since MafA appears to not only regulate insulin expression but also to be involved in insulin secretion [72,75], it is likely that the suppression of MafA protein levels by c-Jun leads to insulin secretory defects that are often observed under diabetic conditions. In conclusion, the augmented expression of c-Jun in diabetic islets decreases MafA activity followed by reduced insulin biosynthesis and secretion, and thereby explains, at least in part, the molecular mechanism for β-cell glucose toxicity that is often observed in type 2 diabetes (Figure 3).

## 3. Involvement of Incretin Signaling in β-Cell Glucose Toxicity

**Figure 4:**
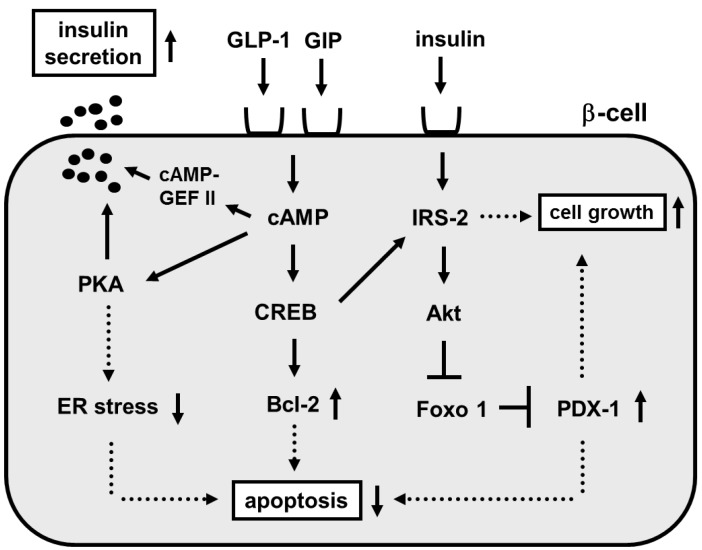
Role of incretin signaling in mature β-cells.

The incretin effect causes more insulin to be secreted when glucose is orally taken compared to when given intravenously even when blood glucose levels have the same profile. This effect is thought to be very important for maximizing insulin response during meals thereby limiting postprandial glucose excursions. Two incretins have been identified: glucagon-like peptide 1 (GLP-1) and glucose-dependent insulinotropic peptide (GIP). It is thought that such incretins play an important role in glucose homeostasis by promoting insulin secretion immediately on meal ingestion. It is well known that incretins (GLP-1 and GIP) bind their incretin receptors (GLP-1 and GIP receptors) in β-cells and increase intracellular cAMP levels, leading to stimulation of insulin secretion through PKA and/or cAMP-GEF II pathways, suppression of β-cell apoptosis through phosphorylation of CREB and increased bcl-2 expression and increase of β-cell growth through phosphorylation of CREB and increased IRS-2 expression (Figure 4).

**Figure 5:**
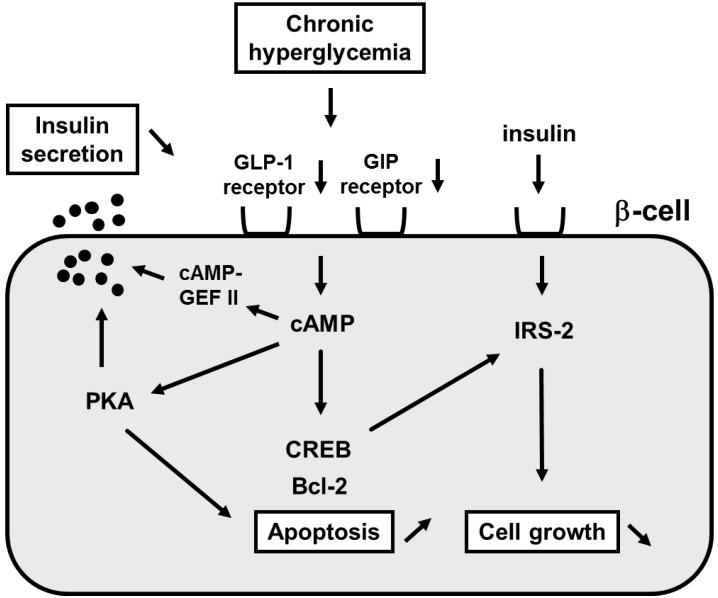
Down-regulation of incretin receptors in pancreatic β-cells under diabetic conditions. Under diabetic conditions, expression of incretin receptors (GLP-1 and GIP receptors) in β-cells are down-regulated, leading to decrease of insulin secretion, increase of β-cell apoptosis and decrease of β-cell growth. Therefore, it is likely that down-regulation of incretin receptor expression is involved in β-cell glucose toxicity found in type 2 diabetes.

Although plasma GLP-1 and GIP levels after meals are almost normal in type 2 diabetes, striking abnormalities are observed in the action of incretin hormones in type 2 diabetes [103]. It was reported that GLP-1 and GIP receptor expression was decreased in a glucose-dependent manner in islets isolated from 90% pancreatectomized diabetic (Px) rats [104]. Such decrease was not observed after normalization of blood glucose levels with phlorizin, which is known to lower blood glucose levels by preventing glucose reabsorption from the glomerular filtrate in the kidney. These results suggest that hyperglycemia *per se* leads to down-regulation of GLP-1 and GIP receptor expression. Furthermore, insulin response to GLP-1 or GIP was markedly reduced in islets isolated from diabetic rats compared to those from control rats [104]. These results indicate that down-regulation of GLP-1 and GIP receptor expression leads to the deterioration of β-cell function. Similar results were reported in obese type 2 diabetic *db*/*db* mice; incretin receptor expression in islets was markedly decreased at 16 weeks of age in *db*/*db* mice but was preserved by normalization of blood glucose levels with insulin therapy [105]. Furthermore, down-regulation of GLP-1 receptor expression was observed in type 2 diabetic subjects as observed in diabetic rodents [106]. These results strengthen the hypothesis that hyperglycemia *per se* leads to down-regulation of GLP-1 and GIP receptor expression. In addition, it has been shown that GLP-1 and GIP expression is down-regulated by lipotoxicity; when islets are exposed to high free fat acids, GLP-1 and GIP expression is decreased [107] (Figure 5). 

It is well known that transcription factor TCF7L2 is closely associated with type 2 diabetes and plays an important role in glucose metabolism [108,109,110,111,112,113,114,115,116]. Human genetics studies have revealed that common variants of the TCF7L2 gene are strongly associated with type 2 diabetes mellitus [108,109,110,111]. Furthermore, recent reports clearly demonstrated that TCF7L2 plays a crucial role in the maintenance of pancreatic β-cell function [113,114,115,116]. Interestingly, it was reported that such TCF7L2 is involved in down-regulation of GLP-1 receptor expression found in diabetes [105]. Indeed, it was shown that expression level of the GLP-1 receptor was lower in isolated human islets treated with siRNA to TCF7L2. Insulin secretion stimulated by glucose or GLP-1 was also impaired in isolated human islets treated with siRNA to TCF7L2 [105]. Furthermore, recent studies have clearly shown that GLP-1 receptor expression level is down-regulated by inactivation or deficiency of TCF7L2 in β-cells [115,116]. Taken together, we think that the down-regulation of incretin receptors by hyperglycemia is largely responsible for the impaired incretin effects and thus, at least in part, explains the molecular mechanism for β-cell dysfunction found in diabetes (Figure 5).
